# Adiponectin Facilitates Postconditioning Cardioprotection through Both AMPK-Dependent Nuclear and AMPK-Independent Mitochondrial STAT3 Activation

**DOI:** 10.1155/2020/4253457

**Published:** 2020-03-04

**Authors:** Qiqi Zhu, Haobo Li, Xiang Xie, Xiaozhen Chen, Ramoji Kosuru, Sisi Li, Qingquan Lian, Chi Wai Cheung, Michael G. Irwin, Ren-shan Ge, Zhengyuan Xia

**Affiliations:** ^1^Department of Anesthesiology, The Second Affiliated Hospital and Yuying Children's Hospital of Wenzhou Medical University, Wenzhou, China; ^2^Department of Anesthesiology, The University of Hong Kong, Hong Kong; ^3^State Key Laboratory of Pharmaceutical Biotechnology, The University of Hong Kong, Hong Kong; ^4^Department of Anesthesiology, Affiliated Hospital of Guangdong Medical University, Zhanjiang, China

## Abstract

Myocardial ischemic postconditioning- (IPo-) mediated cardioprotection against myocardial ischemia-reperfusion (IR) injury needs the activation of signal transducer and activator of transcription 3 (STAT3), which involves adiponectin (APN). APN confers its biological effects through AMP-activated protein kinase- (AMPK-) dependent and AMPK-independent pathways. However, the role of AMPK in APN-mediated STAT3 activation in IPo cardioprotection is unknown. We hypothesized that APN-mediated STAT3 activation in IPo is AMPK-independent and that APN through AMPK-dependent STAT3 activation facilitates IPo cardioprotection. Here, Sprague-Dawley rats were subjected to myocardial IR without or with IPo and/or APN. APN or IPo significantly improved postischemic cardiac function and reduced myocardial injury and oxidative stress, and their combination further attenuated postischemic myocardial injuries. APN or its combination with IPo but not IPo alone significantly increased AMPK activation and both nuclear and mitochondrial STAT3 activation, while IPo significantly enhanced mitochondrial but not nuclear STAT3 activation. In primarily isolated cardiomyocytes, recombined globular APN (gAd), hypoxic postconditioning (HPo), or their combination significantly attenuated hypoxia/reoxygenation-induced cell injury and increased nuclear and/or mitochondrial STAT3 activation. STAT3 inhibition had no impact on gAd or gAd in combination with HPo-induced AMPK activation but abolished their cellular protective effects. AMPK inhibition did not affect HPo cardioprotection but abolished gAd cardioprotection and disabled gAd to facilitate/enhance HPo cardioprotection and STAT3 activation. These results suggest that APN confers cardioprotection through AMPK-dependent and AMPK-independent STAT3 activation, while IPo confers cardioprotection through AMPK-independent mitochondrial STAT3 activation. Joint use of APN and IPo synergistically attenuated myocardial IR injury by activating STAT3 via distinct signaling pathways.

## 1. Introduction

Acute myocardial infarction (AMI) is one of the main causes of morbidity and mortality in coronary heart disease. Timely restoration of the blood flow (reperfusion) remains the mainstay of all current therapeutic approaches to rescue the ischemic myocardium. However, reperfusion may paradoxically exacerbate tissue injury, and this additional damage is called ischemia/reperfusion (IR) injury [1].

Signal transducer and activator of transcription (STAT) 3, a transcription factor that belongs to the STAT family, participates in a wide variety of physiological processes (e.g., proliferation and apoptosis) and protects the hearts against myocardial hypertrophy and myocardial IR injury [2–4]. STAT3 can be activated through phosphorylation at two residues: serine (Ser) 727 and tyrosine (Tyr) 705. When phosphorylated at Ser727, STAT3 translocates into the mitochondria and regulates mitochondrial integrity and biogenesis, resulting in the reduction of reactive oxygen species (ROS) production [5]. However, when phosphorylated at Tyr705, STAT3 translocates into the nucleus where it promotes transcription of cardioprotective genes and improves cellular antioxidant property [6]. Cardiac-specific STAT3 knockout mice demonstrated increased postischemic mortality and cardiac injury following myocardial IR [7]. Moreover, cardiomyocyte-restricted STAT3 deletion rendered the hearts more sensitive to lipopolysaccharide-induced inflammatory damage [8]. All these indicate an important role of STAT3 activation in myocardial protection. Thus, effective means that can activate STAT3 may attenuate myocardial IR injury through concomitantly reducing oxidative stress by improving mitochondrial biogenesis [9] and increasing antioxidant capacity by promoting the expression of nuclear antioxidant genes.

Ischemic postconditioning (IPo), a phenomenon that brief repetitive episodes of ischemia and reperfusion applied at the immediate onset of reperfusion, has been proven to be an effective cardioprotective strategy that protects the hearts against myocardial IR injury [10]. In mice with cardiomyocyte-restricted deletion of STAT3, the threshold of IPo was increased (more cycles of short periods of IR was required for IPo to confer cardioprotection), indicating that the cardioprotective effects of IPo were reduced [11]. Pharmacological inhibition of STAT3 abolished IPo-mediated cardioprotection in pigs with regional myocardial IR [12], while strategies that can enhance STAT3 activation confer cardioprotection both in animal models of myocardial IR injury [13, 14] and in humans [4]. These findings collectively indicate that STAT3 activation plays an essential role in IPo cardioprotection against myocardial IR injury. However, how IPo activates STAT3 to confer cardioprotection remains unclear.

We previously showed that adiponectin (APN), a protein secreted from adipocytes and cardiomyocytes, is essential in IPo-mediated mitochondrial STAT3 activation and the subsequent protection against myocardial IR injury [3]. APN has been shown to reduce liver fibrosis by modulating the Jak-STAT3 signaling pathway which required AMP-activated protein kinase (AMPK) in the liver [15]. Given that AMPK is well known as the main downstream effector in the APN signaling pathway [16], it is possible that AMPK may play a role in APN-mediated STAT3 activation in IPo cardioprotection. Interestingly, a recent study showed that although AMPK activation was increased in IPo-induced cardioprotection, inhibition of AMPK had no impact on IPo-induced cardioprotection [17], suggesting that AMPK may not be the major cellular mediator in IPo cardioprotection. Along with the most recent finding showing that APN-mediated cardioprotection can be AMPK-dependent and AMPK-independent [18], it can be speculated that IPo may activate STAT3 through APN in an AMPK-independent manner. Therefore, we hypothesized that APN may facilitate IPo-mediated AMPK-independent activation of mitochondrial STAT3 and that APN supplementation may provide additional/synergistic effects on IPo cardioprotection by activating nuclear STAT3 in an AMPK-dependent manner.

## 2. Materials and Methods

### 2.1. Animals

Male Sprague-Dawley rats (250 ± 10 g, 6-8 weeks) supplied by the laboratory Animal Service Center (The University of Hong Kong) were used. All rats were housed and given free access to standard rat chow and water in accordance with the principles of Animal Care of the University of Hong Kong. The investigation conformed to the procedures described in the Guide for the Care and Use of Laboratory Animals published by the United States National Institutes of Health (NIH Publication number 85-23, revised 1996). The experimental protocol used in this study was approved by the Wenzhou Medical University Laboratory Animal Ethics Committee and the Committee for Use of Live Animals in Teaching and Research (CULATR) of the University of Hong Kong. Animal experiments were conducted in the Wenzhou Medical University and the University of Hong Kong.

### 2.2. Animal Experimental Protocol

Rats were randomly divided into five groups: (1) sham-operated group (group sham); (2) rats subjected to myocardial IR (group IR); (3) rats injected with one dose of recombinant APN adenovirus (1 × 10^9^ pfu, tail vein injection) 7 days before being subjected to myocardial IR (group IR+APN), as previously described [19]; (4) rats subjected to myocardial IR with IPo achieved by inducing three cycles of 10 seconds of reocclusion and 10 seconds of reperfusion at the onset of reperfusion (group IPo); and (5) rats injected with recombinant APN adenovirus 7 days before myocardial IR and then received IPo (group IPo+APN).

### 2.3. *In Vivo* Model of Myocardial Ischemic Reperfusion and Ischemic Postconditioning

After anesthesia by intraperitoneal injection of sodium pentobarbital (65 mg/kg), animals were randomized to receive sham operation, myocardial IR, or IPo. Myocardial IR was induced by temporarily exteriorizing the heart via a left thoracic incision and occluding the left anterior descending (LAD) coronary artery 30 minutes (min) followed by reperfusion for 2 hours [3, 20]. IPo was produced by three cycles of 10 seconds of reocclusion and 10 seconds of reperfusion immediately after the completion of ischemia [3]. At the completion of 2 hours of reperfusion, left ventricular tissue was harvested and immediately frozen in liquid nitrogen until analyzed.

### 2.4. Measurement of Left Ventricular Function

Hemodynamics were continuously monitored using subcutaneous stainless-steel electrodes that were connected via a cable to a PowerLab monitoring system (ML750 PowerLab/4sp with MLT380 Reusable BP Transducer; AD Instruments, CO Springs, CO). Heart rate, mean arterial pressure (MAP), and the rate pressure product (RPP, the product of heart rate and systolic pressure) were recorded. The RPP was calculated to evaluate the myocardial oxygen demand of the animals [21].

### 2.5. Determination of Myocardial Infarct Size

At the end of reperfusion, myocardial infarct size was measured using TTC (1% 2,3,5-triphenyltetrazolium chloride) staining as described [21] and the operators were initially blinded to the information of study design and intervention. Briefly, the LAD was reoccluded and cannulated just distal to the occlusion site. One milliliter (mL) of 5% Evans Blue was intravenously administrated. The stained region of the heart was identified as the normal region without ischemia-reperfusion episodes. The unstained region was considered as the area-at-risk (AAR). The heart was immediately fibrillated, removed, and sliced into serial transverse sections 6 to 7 mm in width and incubated in 1% TTC buffer for 30 min at room temperature. The heart slices were then fixed with 10% fresh formalin for 24 hours. The area unstained by TTC was identified as the infarcted tissue. Myocardial infarct size was expressed as a percentage of the AAR.

### 2.6. Measurement of Plasma Creatinine Kinase-MB Levels

Creatinine kinase-MB (CK-MB) isoenzyme is a major biomarker for myocardial cellular injury. After 2 hours of reperfusion, blood samples were collected for measurement of CK-MB by enzyme immunoassay using a commercial kit (Cloud-Clone Corp, Houston, TX) as described [21].

### 2.7. Apoptotic Cell Death Detection Using Terminal Deoxynucleotidyl Transferase dUTP Nick-End Labeling

Terminal deoxynucleotidyl transferase dUTP nick-end labeling (TUNEL) reaction was performed using an *in situ* cell death detection kit (Roche Supplementary Diagnostics GmbH, Mannheim, Germany) as previously described [3]. The sections were observed in the fluorescent microscope by an investigator, who was initially blinded to treatment groups, and five randomly selected fields of each slide were analyzed, and the apoptotic index was calculated as a percentage of apoptotic nuclei to total nuclei.

### 2.8. Adult Rat Ventricular Cardiomyocyte Isolation and Hypoxia/Reoxygenation

Calcium-tolerant cardiomyocytes were isolated from rat ventricles via a modified method as previously described [22]. Rats were sacrificed with an intraperitoneal injection of overdose sodium pentobarbital (220 mg/kg) and heparinized. The hearts were rapidly removed and mounted on a Langendorff perfusion apparatus and proceeding to cardiomyocyte isolation as we described [3]. Cells isolated from a single rat heart were plated onto Matrigel-coated culture dishes and allowed to recover for 3 hours. Cultured ventricular cardiomyocytes were incubated in Medium 199 (Gibco, Grand Island, NY) at 37°C.

Cardiomyocytes were, respectively, treated with stattic (a specific STAT3 inhibitor; 100 mmol/L, 10 min), Dorsomorphin (also named compound C, CC, a specific AMPK inhibitor; 5 *μ*mol/L, 1 hour), or recombinant globular APN (gAd) (2 *μ*mol/L, 24 hours) [3] before being subjected to hypoxia/reoxygenation (HR) and hypoxic postconditioning (HPo). Stattic, the first nonpeptidic small molecule, potently inhibits STAT3 activation and nuclear translocation with IC50 of 5.1 *μ*M in cell-free assays. Dorsomorphin (compound C) is a potent, reversible, and selective AMPK inhibitor with Ki of 109 nM in cell-free assays, which exhibits no significant inhibition for several structurally related kinases including ZAPK, SYK, PKA, and JAK3.

HR was achieved by subjecting the cells to hypoxia for 45 min followed by 2 hours of reoxygenation. Hypoxia conditions were obtained by equilibrating a humidified Plexiglas chamber containing myocytes with 95% N_2_ and 5% CO_2_ and confirmed by measuring the chamber O_2_ concentration falling to 0.1%. Reoxygenation was achieved by exposing cells to room air. HPo was achieved by three cycles of 5 min of reoxygenation and 5 min of hypoxia before prolonged reoxygenation [3]. At the end of treatments, cells were fixed for immunofluorescence staining or collected and snap-frozen in liquid nitrogen for future analysis, as described below. Experiments were repeated three times and each in duplicate.

### 2.9. Measurement of Cellular ROS in Cultured Cardiomyocyte

Superoxide generation in cultured cardiomyocytes was estimated by dihydroethidium (DHE) staining as previously described [23, 24]. Briefly, cardiomyocytes were loaded with DHE at a concentration of 10 *μ*M for 30 min at 37°C. The DHE fluorescence of DHE-labeled positive nuclei was calculated in each of five randomly selected fields and was expressed as a percentage of the DHE-stained positive myocyte nuclei compared with control by a quantitative morphometric method.

### 2.10. Determination of Cellular Injury

Cell apoptosis was detected by double immunofluorescence staining of TUNEL, using the *In Situ* Cell Death Detection Kit (Roche, Indianapolis, IN, USA), and cell lactate dehydrogenase (LDH) content was measured with an LDH Cytotoxicity Assay Kit (Roche, Indianapolis, IN, USA) as described [25].

### 2.11. Separation of Myocardial Cytosolic and Nuclear Fractions and Isolation of Mitochondria

At the end of 2 hours of postischemic reperfusion, heart tissues were immediately collected for separation of cytosolic and nuclear fractions and for isolation of mitochondria. Hearts were cleared of blood by washing thoroughly in Tyrode buffer and aortic and atrial sections removed from the ventricles. Ventricular tissue was freeze-clamped in liquid nitrogen and stored until fractionated to isolate cytosolic and nuclear fractions (Nuclear and Cytoplasmic Extraction Kit, Thermo, Chicago, IL) and mitochondria (Mitochondria Extraction Kit, Thermo, Chicago, IL) according to the manufacturer's protocol as described [3].

### 2.12. Extraction of Total RNA and Quantitative Real-Time Polymerase Chain Reaction Analysis

Total RNA was extracted using Trizol (Invitrogen Life Technologies, Carlsbad, CA), equal amounts of RNA were reverse-transcribed and processed using the PrimeScript RT Master Mix Kit, and quantitative real-time polymerase chain reaction (PCR) was performed with an SYBR green PCR master mix (Takara) on a 7300 ABI-Prizm Sequence Detector (Applied Biosystems) as described [19], for measurement of superoxide dismutase 1 (SOD1, MnSOD), nuclear factor erythroid 2-related factor (Nrf) 2, heme oxygenase 1 (HO-1), Nrf1, and peroxisome proliferator-activated receptor-*γ* coactivator-1*α* (PGC-1*α*) genes with *β*-Actin as reference. The conditions for amplification were 30 seconds at 95°C for denaturation, followed by 40 cycles of 5 seconds at 95°C and 30 seconds at 60°C. Gene-specific primers were as follows: SOD1 forward, 5′-AACCAGTTGTGGTGTCAGGA-3′, reverse, 5′-CTCCTGAGAGTGAGATCACA-3′; catalase forward, 5′-TTCTACACTGAAGATGGTAACTG-3′, reverse, 5′-GAAAGTAACCT GATGGAGAGAC-3′; Nrf2 forward, 5′-GAATAAAGTTGCCGCTCAGAA-3′, reverse, 5′-AA GGTTTCCCATCCTCATCAC-3′; HO-1 forward, 5′-TGCTCGCATGAACACTCTG-3′, reverse, 5′-TCCTCTGTCA GCAGTGCCT-3′; Nrf1 forward, 5′-GATGCTTCAGAACTGCCAACCA-3′, reverse, 5′-GGTC ATTTCACCGCCCTGTAAC-3′; PGC-1*α* forward, 5′-CACTGACAGATGGA GCCGTGA-3′, reverse, 5′-TGTTGGCTGGTGCCAGTAAGAG-3′; and *β*-Actin, served as an endogenous control, forward, 5′-AGGCCAACCGTGAAAAGATG-3′, reverse, 5′-ACCAGAGG CATACAGGGACA A-3′. The level of target gene expression was analyzed using the 2(-*Δ*ΔCT) method and normalized against the *β*-actin gene.

### 2.13. Western Blot Analysis for the Protein Expression and Signaling Proteins

Equal protein amounts from isolated cardiomyocytes, rat heart, and isolated mitochondria were resolved by 8-12% SDS-PAGE and transferred to polyvinylidene fluoride membrane for immunoblot analysis, as previously described [26]. Membranes were blocked with 5% nonfat milk in Tris-Buffered Saline- (TBS-) Tween and were incubated with primary antibodies overnight at 4°C at the following dilutions: AMPK, phosphorylated AMPK (Thr172), STAT3, phosphorylated STAT3 at Tyr705 (p-STAT3 Tyr705), Bax, Bcl2, caspase-3, and cleaved caspase-3 (Cell Signaling Technology, Beverly, MA) 1 : 1000; phosphorylated STAT3 at Ser727 (p-STAT3 Ser727) (Cell Signaling Technology, Beverly, MA) 1 : 500. After washing with TBS-Tween, immunoreactive bands were visualized by an enzymatic chemiluminescence method and quantified with Quantity One image software.

### 2.14. Statistical Analysis

All the values were expressed as mean ± standard error of the mean (SEM). One-way analysis of variance (ANOVA) was used for statistical analyses (GraphPad Prism, USA) of data obtained within the same group and between groups, respectively, followed by Tukey's test for multiple comparisons of group means. A *p* < 0.05 was considered statistically significant.

## 3. Results

### 3.1. Adiponectin-Activated Posthypoxic Cardiomyocyte Nuclear STAT3 and Mitochondrial STAT3, Respectively, via AMPK-Dependent and AMPK-Independent Pathways

**Figure 1:**
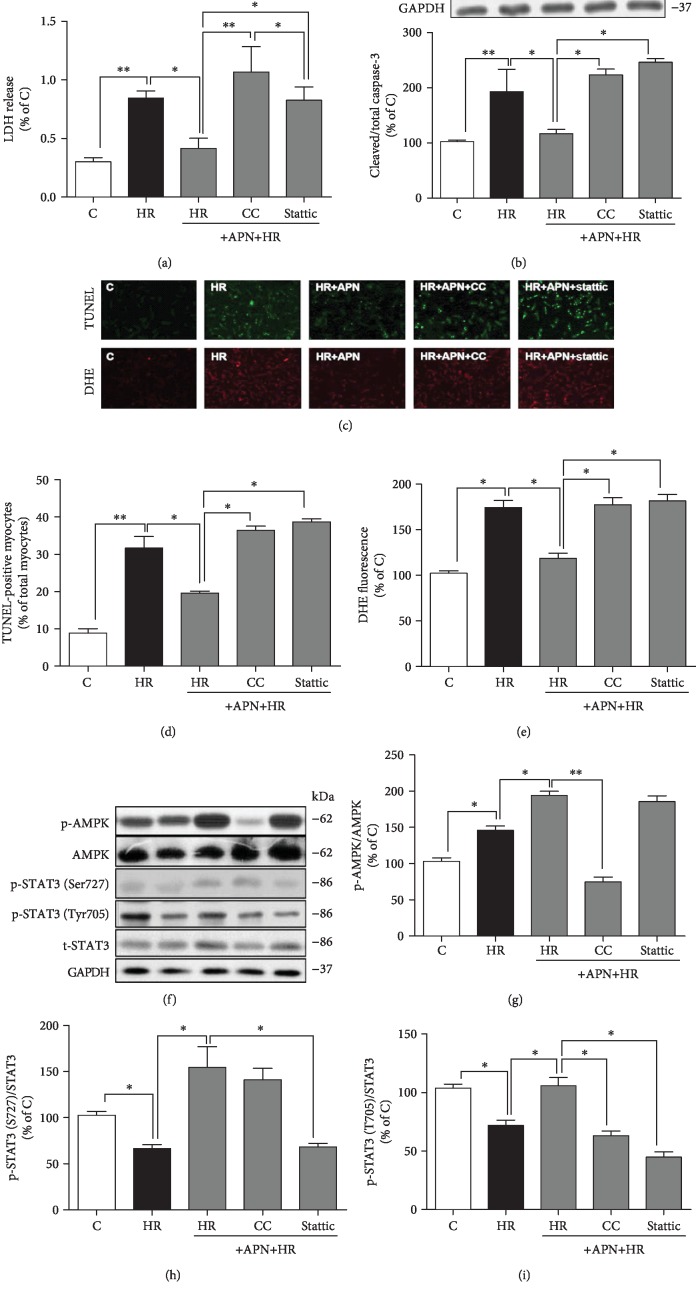
Adiponectin activated posthypoxic cardiomyocyte nuclear STAT3 and mitochondrial STAT3, respectively, via AMPK-dependent and AMPK-independent pathways. Isolated cardiomyocytes were subjected to hypoxia-reoxygenation (HR) with globular APN (gAd) in the absence or presence of AMPK inhibitor compound C (CC) or STAT3 inhibitor stattic. (a) Cardiomyocyte death assessed by lactate dehydrogenase (LDH) release. (b) Protein expression of cleaved and total caspase-3. (c) Representative images for TUNEL staining and dihydroethidium (DHE) staining. (d) Cell apoptosis assessed by TUNEL staining. (e) Cellular reactive oxygen species production assessed by DHE staining. (f) Representative images for western blotting. (g–i) Protein expression of phosphorylated and total AMPK, STAT3 (Ser727), and STAT3 (Tyr705). Data are mean ± SEM of two independent experiments each performed in triplicate: ^∗^*p* < 0.05 and ^∗∗^*p* < 0.01.

As shown in Figure 1, in cultured isolated cardiomyocytes, posthypoxic cell death was significantly increased, manifested as elevated LDH release, enhanced protein expression of cleaved caspase-3 (marker of cell apoptosis), and increased TUNEL-positive cells (Figures 1(a)–1(e)), which were associated with enhanced ROS evidenced by increased number of DHE-labeled positive nuclei (Figures 1(c) and 1(e)). All these changes were attenuated or prevented by globular APN (gAd) supplementation (Figures 1(a)–1(e)). However, these protective effects of gAd were cancelled by either AMPK inhibition with the specific inhibitor compound C (CC) or by STAT3 inhibition with the specific inhibitor stattic (Figures 1(a)–1(e)). HR induced a significant increase of AMPK phosphorylation/activation (p-AMPK), which was concomitant with significant reductions of STAT3 phosphorylation at both Ser727 and Tyr705, while gAd treatment further enhanced p-AMPK and reversed HR-induced reductions of STAT3 phosphorylation at both Ser727 and Tyr705 (Figures 1(f)–1(i)). Interestingly, AMPK inhibition cancelled gAd-induced activation of STAT3 at Tyr705 but not at Ser727 (Figures 1(h) and 1(i)), while STAT3 inhibition had no effect on gAd-induced AMPK activation (Figure 1(g)), suggesting that gAd-induced STAT3 activation at Tyr705 is AMPK-dependent while gAd-induced mitochondrial STAT3 activation at Ser727 is AMPK-independent.

### 3.2. Hypoxic Postconditioning (HPo) Enhanced Mitochondrial STAT3 (Ser727) Activation but Not Nuclear STAT3 (Tyr705) Activation Independent of AMPK Activation

**Figure 2:**
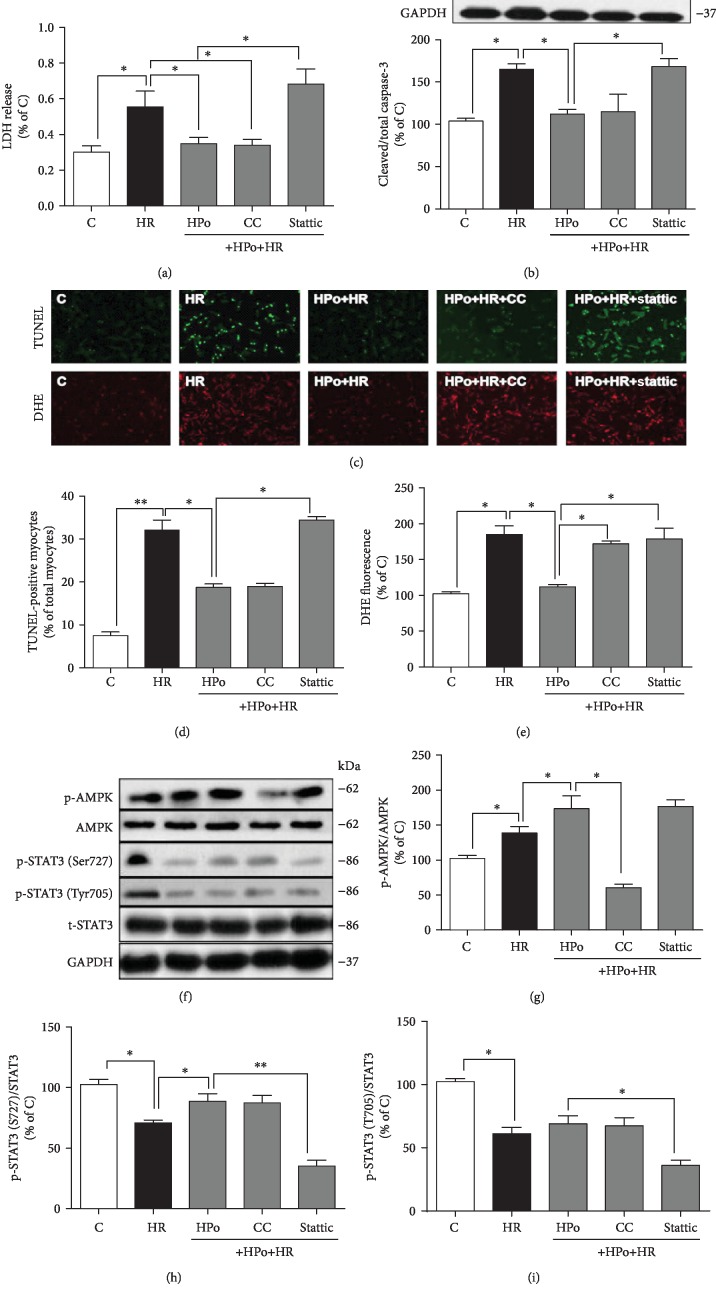
Hypoxic postconditioning (HPo) enhanced mitochondrial STAT3 (Ser727) but not nuclear STAT3 (Tyr705) independent of AMPK activation. Isolated cardiomyocytes were subjected to hypoxia-reoxygenation (HR) with hypoxic postconditioning (HPo) in the absence or presence of AMPK inhibitor compound C (CC) or STAT3 inhibitor stattic. (a) Cardiomyocyte death assessed by lactate dehydrogenase (LDH) release. (b) Protein expression of cleaved and total caspase-3. (c) Representative images for TUNEL staining and dihydroethidium (DHE) staining. (d) Cell apoptosis assessed by TUNEL staining. (e) Cellular reactive oxygen species production assessed by DHE staining. (f) Representative images for western blotting. (g–i) Protein expression of phosphorylated and total AMPK, STAT3 (Ser727), and STAT3 (Tyr705). Data are mean ± SEM of two independent experiments each performed in triplicate: ^∗^*p* < 0.05 and ^∗∗^*p* < 0.01.

In parallel to HR-induced significant cell injury (Figures 2(a)–2(e)), the phosphorylation of STAT3 at both Tyr705 and Ser727 (Figures 2(f), 2(h), and 2(i)) was significantly reduced despite a significant increase of AMPK activation (Figure 2(g)). This HR-induced cellular injury was attenuated by HPo (Figures 2(a)–2(e)) with concomitant enhancement of STAT3 activation at Ser727 (Figure 2(h)) but not at Tyr705 (Figure 2(i)). STAT3 inhibition reverted HPo-mediated attenuation of cellular LDH release and cell apoptosis and the HPo-mediated attenuation of oxidative stress (Figures 2(a)–2(e)), while AMPK inhibition had no impact on HPo-mediated reduction of apoptosis but cancelled HPo-mediated reduction of oxidative stress (Figures 2(c) and 2(e)). Posthypoxic AMPK activation was moderately but significantly increased that was concomitant with reduced STAT3 activation at both phosphorylation residues (Figures 2(f)–2(i)). HPo treatment further significantly enhanced posthypoxic AMPK activation and increased posthypoxic STAT3 activation at Ser727 but not at Tyr705 (Figure 2(i)). AMPK inhibition had no impact on HPo-induced STAT3 activation (Figures 2(h) and 2(i)). Similarly, STAT3 inhibition had no significant effect on HPo-induced AMPK activation (Figure 2(g)). This suggests that HPo-induced mitochondrial STAT3 (Ser727) activation is AMPK-independent.

### 3.3. Adiponectin Provided Additive Cardioprotective Effects to Ischemic Postconditioning (IPo) *In Vivo*

**Figure 3:**
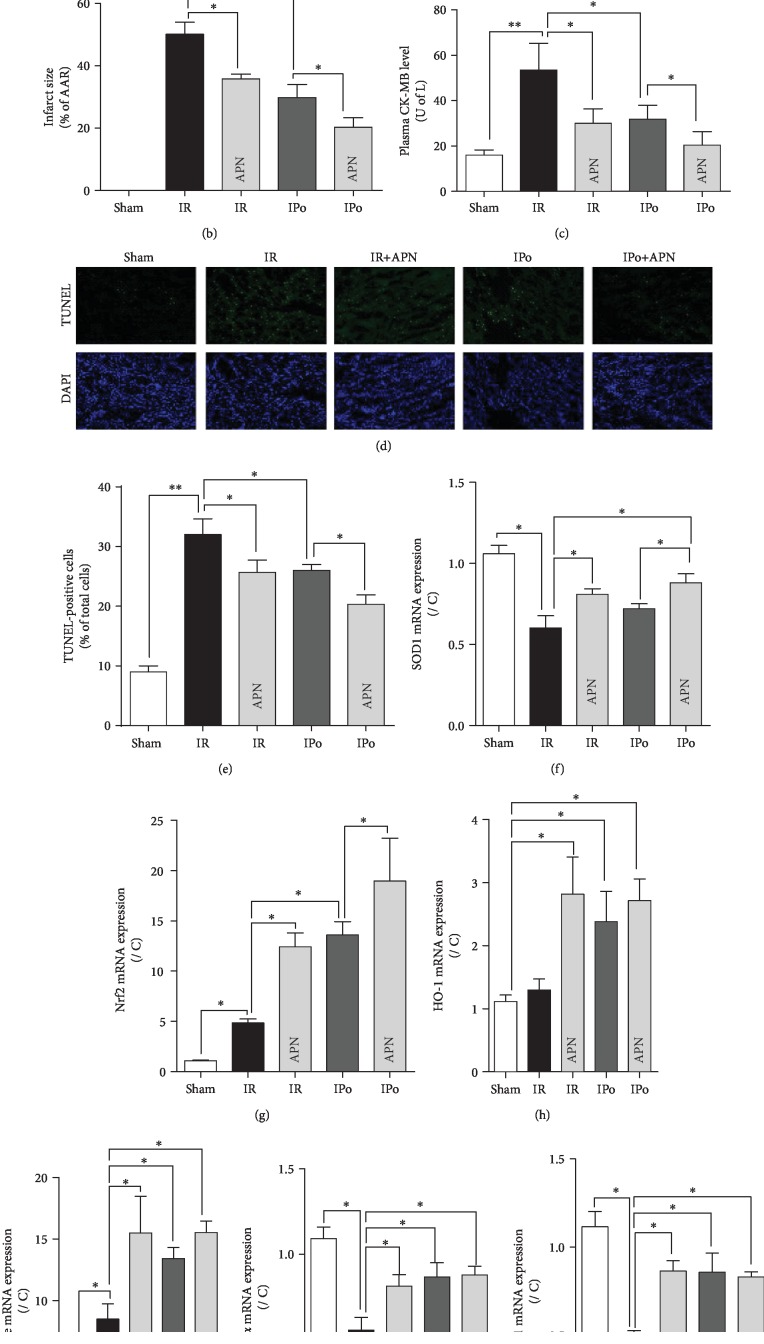
Adiponectin provided additive cardioprotective effects to ischemic postconditioning. Rats were subjected to myocardial ischemia-reperfusion (IR) in the absence or presence of APN (1 × 10^9^ pfu), ischemic postconditioning (IPo), or their combination (APN+IPo). (a, b) Myocardial infarct size determined by TTC staining and expressed as a percentage of the area-at-risk (AAR) served by the occluded artery. (c) Plasma level of creatine kinase- (CK-) MB. (d, e) Cell apoptosis assessed by TUNEL staining. (f–k) mRNA expression of SOD1, Nrf2, HO-1, catalase, PGC-1*α*, and Nrf1. Data are mean ± SEM, with *n* = 8 animals *per* group: ^∗^*p* < 0.05 and ^∗∗^*p* < 0.01.

Adiponectin supplementation at the dose used did not cause animal death. As shown in Table 1 and Figure 3, in rats subjected to *in vivo* myocardial IR, postischemic cardiac function was compromised, manifested as reduced heart rate, MAP, and RPP that was associated with myocardial injury evidenced by increased postischemic infarct size, elevated plasma CK-MB release, and enhanced myocardial cell apoptosis manifested as increased TUNEL-positive cells (Figures 3(a)–3(e)). Either APN or IPo treatment significantly attenuated the impairment in postischemic cardiac function and reduced myocardial injuries, while APN in combination with IPo conferred superior protective effects than either APN or IPo alone manifested as more significant reductions in postischemic infarct size, plasma CK-MB, and apoptosis (Figures 3(a)–3(e)). Myocardial IR significantly reduced SOD1 (Cu-Zn-SOD) (Figure 3(f)) but increased Nrf2 (Figure 3(g)) and catalase (Figure 3(i)) mRNA expression in the nuclear that were associated with decreased PGC-1*α* and Nrf1 mRNA expression in the mitochondria (Figures 3(j) and 3(k)), while nuclear HO-1 mRNA expression remained unchanged (Figure 3(h)). Either APN or IPo significantly enhanced SOD1, Nrf2, HO-1, catalase, PGC-1*α*, and Nrf1 mRNA expression (Figures 3(f)–3(k)), while APN further significantly potentiated IPo-induced enhancements of SOD1 and Nrf2 mRNA expression.

### 3.4. Adiponectin and Ischemic Postconditioning Exerted Distinct Effects on Cardiac AMPK and STAT3 Activation *In Vivo*

**Figure 4:**
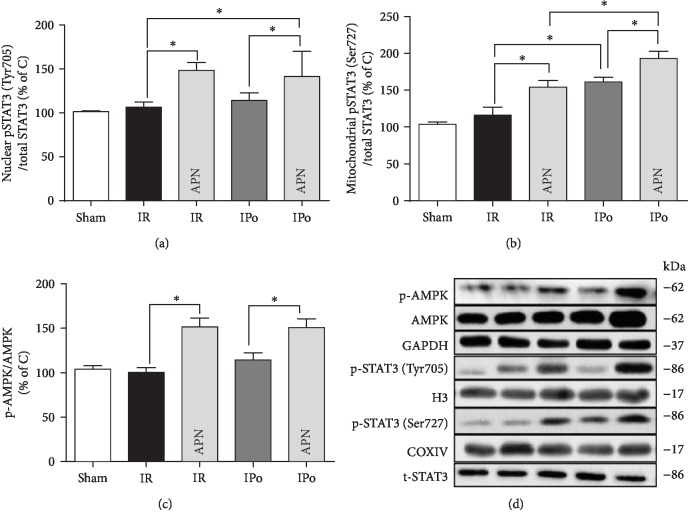
Adiponectin and ischemic postconditioning exerted distinct effects on cardiac AMPK and STAT3 activation *in vivo*. Rats were subjected to myocardial ischemia-reperfusion (IR) in the absence or presence of APN (1 × 10^9^ pfu), ischemic postconditioning (IPo), or their combination (APN + IPo). (a) Protein expression of nuclear phosphorylation (at Tyr705) and total STAT3. (b) Protein expression of mitochondrial phosphorylated (at Ser727) and total STAT3. (c) Protein expression of phosphorylated and total AMPK. (d) Representative images of western blotting. Data are mean ± SEM, with *n* = 8 animals *per* group: ^∗^*p* < 0.05 or 0.01.

As shown in Figure 4, postischemic myocardial STAT3 activation (at Tyr705 and Ser727) was slightly increased but this increase did not reach statistical significance (Figures 4(a) and 4(b)). APN significantly increased the activation of posthypoxic cardiac STAT3 at both residues (Ser727 and Tyr705) and the activation of AMPK (Figures 4(a)–4(d)) either in the absence or presence of IPo. IPo alone significantly enhanced STAT3 activation at Ser727 but had no impact on Tyr705 nor did it affect AMPK activation (Figures 4(a)–4(d)). APN and IPo combination resulted in a further increase in STAT3 activation at Ser727 when compared to either APN or IPo alone (Figure 4(b)).

### 3.5. STAT3 Inhibition Abolished While AMPK Inhibition Compromised the Cardioprotection Conferred by Adiponectin in Combination with Hypoxic Postconditioning

**Figure 5:**
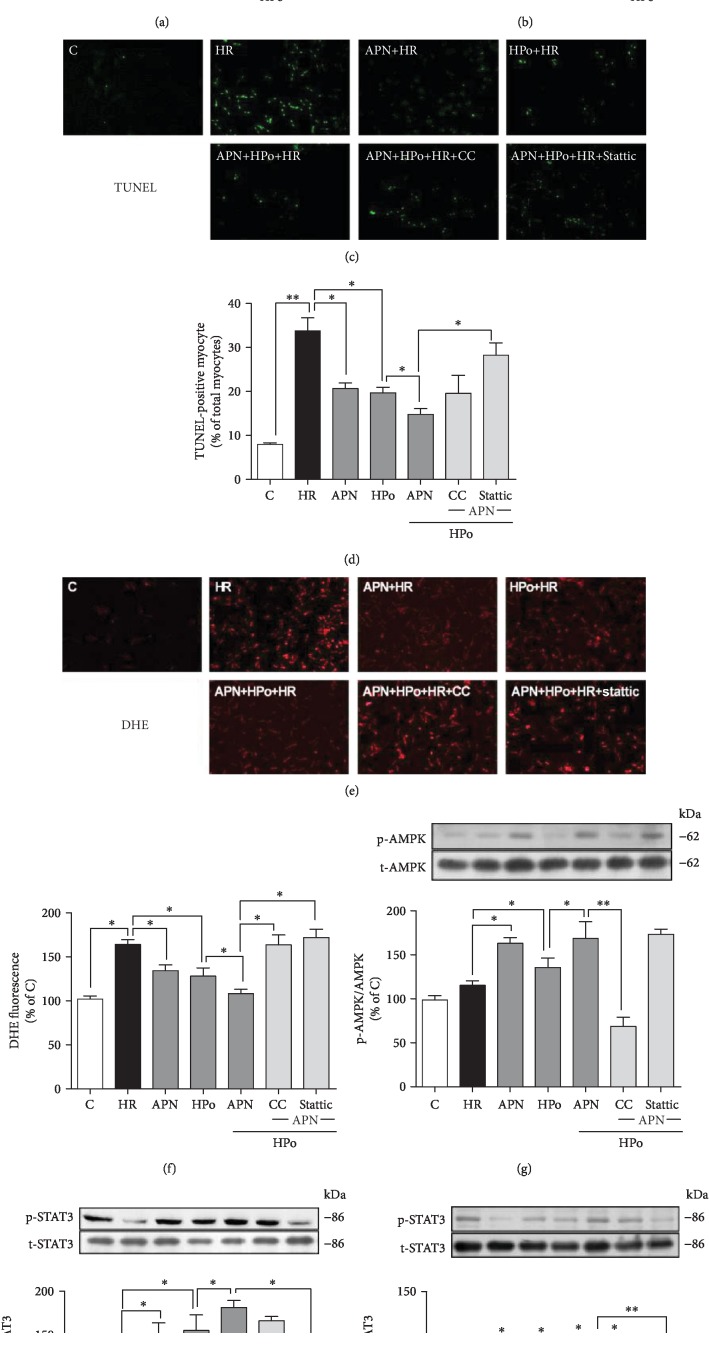
STAT3 inhibition abolished while AMPK inhibition partially reduced cardioprotection induced by adiponectin in combination with hypoxic postconditioning. Isolated cardiomyocytes were subjected to hypoxia-reoxygenation (HR) with globular APN (gAd) or hypoxic postconditioning (HPo), or their combination in the absence or presence of AMPK inhibitor compound C (CC) or STAT3 inhibitor stattic. (a) Cardiomyocyte death assessed by lactate dehydrogenase (LDH) release. (b) Protein expression of cleaved and total caspase-3. (c, d) Cell apoptosis assessed by TUNEL staining. (e, f) Cellular reactive oxygen species production assessed by and dihydroethidium (DHE) staining. (g–i) Protein expression of phosphorylated and total of AMPK, STAT3 (Ser727), and STAT3 (Tyr705). Data are mean ± SEM of two independent experiments each performed in triplicate: ^∗^*p* < 0.05 and ^∗∗^*p* < 0.01.

In order to determine the role of AMPK and STAT3 in the cardioprotection of APN in combination with IPo, primarily isolated cardiomyocytes were subjected to HR and treated with APN and HPo in the absence or presence of the AMPK inhibitor CC or the STAT3 inhibitor stattic. As shown in Figure 5, in cultured cardiomyocytes, the posthypoxic cellular injuries were significantly attenuated by either APN or HPo treatment while APN in combination with HPo conferred superior protective effects to either APN or HPo alone (Figures 5(a)–5(f)). The protective effects of APN in combination with HPo in reducing posthypoxic LDH release and ROS production were reverted by either AMPK inhibition or STAT3 inhibition (Figures 5(a)–5(f)), while STAT3 inhibition but not AMPK inhibition significantly compromised APN in combination with HPo-mediated attenuation of posthypoxic apoptotic cell death (Figure 5(d)). Posthypoxic AMPK activation was significantly increased while STAT3 activation was reduced at both Ser727 and Tyr705 (all *p* < 0.05, group HR *vs.* group c, Figures 5(g)–5(i)). APN alone or its combination with HPo significantly increased the activation of AMPK and the activation of STAT3 at both phosphorylation residues (Figures 5(g)–5(i)), and APN in combination with HPo induced more significant increase of STAT3 activation at Ser727 than APN alone (*p* < 0.05, group APN+HPo *vs.* group APN, Figure 5(h)), while HPo alone enhanced AMPK activation and STAT3 activation at Ser727 but had no impact on STAT3 activation at Tyr705 (Figures 5(g) and 5(i)). AMPK inhibition cancelled the AMPK activation and the activation of STAT3 at Tyr705 induced by combinational treatment with APN and HPo (Figures 5(g)–5(i)), while STAT3 inhibition cancelled APN and HPo-induced STAT3 activation but had no effect on AMPK activation induced by combinational treatment with APN and HPo (Figures 5(g)–5(i)).

## 4. Discussion

Findings from our *in vitro* and *in vivo* studies provided strongly suggest that APN confers cardioprotection through AMPK-dependent nuclear STAT3 activation and AMPK-independent mitochondrial STAT3 activation, while IPo confers cardioprotection mainly through mitochondrial STAT3 activation which is AMPK-independent. APN and IPo synergistically attenuated myocardial IR injury by activating STAT3 via distinct signaling pathways despite that they also share common mechanisms such as both APN and IPo can activate p-STAT3 at Ser727. In isolated primary cardiomyocytes, supplementation of APN attenuated HR-induced oxidative stress and cellular injury, which were reduced by AMPK inhibition or abolished by STAT3 inhibition. APN supplementation increased the activation of AMPK and the activation of STAT3 at both phosphorylation residues, but AMPK inhibition cancelled APN-induced STAT3 activation at Tyr705 but not Ser727, while STAT3 inhibition had no effect on APN-induced AMPK activation, suggesting that APN conferred cardioprotection through AMPK-dependent STAT3 activation at Tyr705 and AMPK-independent STAT3 at Ser727. HPo increased p-AMPK and activated STAT3 at Ser727 but not Tyr705, while AMPK inhibitor CC, at the concentration used, had no effect on HPo-induced STAT3 activation, indicating that HPo conferred cardioprotection mainly through activating STAT3 at Ser727 which is AMPK-independent. Collectively, we conclude that APN and IPo attenuate myocardial IR by activating STAT3 via distinct signaling pathways (i.e., AMPK-dependent and AMPK-independent).

Oxidative stress resulting from the excessive production of ROS and/or inadequate antioxidant defense is the main cause of myocardial IR injury [27–30]. In the current study, we showed that either APN, IPo, or their combination reduced post-ischemic oxidative stress and cardiac injury, which were abolished by STAT3 inhibition, pointing out the importance of STAT3 in APN and IPo-mediated reduction of oxidative stress to confer their cardioprotection. STAT3 is activated via the tyrosine phosphorylation (Tyr705) and serine phosphorylation (Ser727) cascades [6]. When being phosphorylated at Tyr705, STAT3 translocates into the nucleus where STAT3 promotes transcription of antioxidant genes (i.e., Mn-SOD, Nrf2, and HO-1) and exerts its antioxidant effects [31]. When being phosphorylated at Ser727, STAT3 shuttles into the mitochondria primarily where it interacts with Complex I and maintains mitochondrial biogenesis thereby reduces ROS production [5]. In the current study, APN alone increased STAT3 activation at both phosphorylation residues (Ser727 and Tyr705) thereby activated the downstream effectors that target antioxidant molecules in the nucleus (i.e., SOD1, Nrf2, HO-1, and catalase) and in the mitochondria (Nrf1 and PGC-1*α*), while IPo alone enhanced STAT3 activation only at Ser727 and activated Nrf2, HO-1, and catalase in the nucleus and Nrf1 and PGC-1*α* in the mitochondrial but had no effect on SOD1. These findings not only suggest that different regulatory patterns of STAT3 activation in APN and IPo cardioprotection but may also provide a clue that APN through activating STAT3 at Tyr705 facilitates IPo cardioprotection against myocardial IR injury. Indeed, our study demonstrated that combined use of APN and IPo led to further increase of STAT3 activation at both residues and further increase of Nrf2 in the nucleus and conferred superior cardioprotection to either APN or IPo alone. This suggests that maximal activation of STAT3 (phosphorylation at two residues, Ser727 and Tyr705) credits superior cardioprotection by integrating the two distinct pathways, i.e., by promoting nuclear antioxidant genes transcription and by reducing mitochondrial ROS production, while partial activation of STAT3 (Ser727 or Tyr705) favors stimulation of either of the above-mentioned pathways.

Despite the proven importance of STAT3 activation in cardioprotective interventions (e.g., APN and IPo), it is still unclear how APN and IPo activate STAT3 to confer cardioprotection. AMPK is well known as the downstream effector of APN biological function [32–34], and a tight regulatory effect of AMPK on STAT3 activation was evidenced by the findings that up-regulation of AMPK by APN or by specific agonist-attenuated fibrosis and inflammatory response in the liver through inhibition of STAT3 activation [15, 35]. Furthermore, pharmacological activation of AMPK can inhibit STAT3 activation and consequently inhibit monocyte-to-macrophage differentiation and attenuate angiotensin-II-induced atheromatous plaque formation [36]. However, the direct regulatory effect of AMPK on STAT3 in the heart, particularly in the setting of APN and IPo, has not been reported. We found that, in cultured cardiomyocytes, AMPK inhibition cancelled APN-induced STAT3 activation at Tyr705 but not at Ser727 and abolished APN-mediated protection against post-hypoxic cellular injury, indicating that AMPK is involved in the APN-mediated Tyr705 STAT3 activation while APN-mediated Ser727 STAT3 activation is AMPK-independent. Our results are consistent with the findings of previous studies demonstrating that APN cardioprotection is partially but not completely mediated by AMPK [33, 37], which involved the AMPK-independent pathway [18]. This was further confirmed by our current results, showing that inhibition of STAT3 activation at both residues led to a further exacerbation of myocardial injury than that of AMPK inhibition in APN-mediated cardioprotection. The findings indicate that an AMPK-independent pathway exists in APN-induced STAT3 activation and support the notion that STAT3 activation may work as the end-effector in APN cardioprotection [6].

Oxidative stress-induced cardiomyocyte apoptotic cell death is the main cause of post-ischemic cardiac injury. In the current study, we found that either APN or IPo or their combination reduced post-ischemic cell apoptosis *in vivo* in the heart and *in vitro* in cultured cardiomyocytes, while these anti-apoptotic effects of APN or IPo or their combination were abolished by STAT3 inhibition, suggesting that STAT3 activation plays an essential role in their anti-apoptotic effects. Interestingly, in IPo cardioprotection, we found that AMPK was activated by IPo while AMPK inhibition cancelled IPo-induced reduction of oxidative stress but had no impact on IPo-mediated STAT3 activation and the subsequent reduction of cell apoptosis. This finding may be partially explainable by the notion that there exists reactive oxygen species-independent apoptosis in reperfusion injury [38]. Alternatively, differences might exist in the extent of ROS decrease and/or the decrease might occur in different compartments and some of which could be more relevant for causing cell injury. Further, this finding not only suggests that AMPK-mediated reduction of oxidative stress in IPo may not be efficient to confer anti-apoptotic effects but also indicates that AMPK may not be the principal mechanism in IPo cardioprotection. This was in contrast to the recent finding by Hu et al. [39] who demonstrated that AMPK inhibition cancelled IPo-mediated reduction of oxidative stress and attenuation of myocardial injury in neonatal rat cardiomyocytes subjected to HR. The inconsistencies of these results may be due to (1) difference of cell models (cardiomyocytes isolated from adult rats in our study *vs.* cardiomyocytes isolated from neonatal rats in Hu's study), (2) variation of HR models (45 min hypoxia with 2 hours reoxygenation in our study *vs.* 3 hours hypoxia with 6 hours reoxygenation in Hu's study), and (3) variation in AMPK inhibition by compound C (1 hour before hypoxia in our study *vs.* 6 hours after hypoxia in Hu's study). However, these findings together with our and others' previous finding that APN-mediated STAT3 activation is essential in IPo cardioprotection [3] and that APN may exert its biological effects through the AMPK-independent pathway [33] collectively suggest that APN regulates STAT3 activation through AMPK-independent pathway in IPo cardioprotection. More importantly, this provides the possibility that APN may act through AMPK-dependent STAT3 activation to facilitate IPo cardioprotection. Thus, effective means that can activate APN signaling pathways (such as the newly identified APN receptor agonist [40] and N-Acetylcysteine [21]) may provide additional cardioprotection on top of IPo or rescue IPo cardioprotection in combating myocardial IR injury, in particular, under conditions that IPo cardioprotection is diminished or abolished such as in hearts from subjects with diabetes or the elderly [3]. It should be noted that despite of the obvious synergistic cardioprotective effects observed between APN and IPo in an *in vivo* model of myocardial IR, mechanistic explorations in our current study were mainly conducted in isolated cultured cardiomyocytes that have excluded the potential impacts of systemic factors such as hormonal changes that may be induced by APN and/or IPo *in vivo* and the potential interactions of different type of cells such as cardiomyocytes and vascular endothelial cells. Nevertheless, current studies shall stimulate further studies to be conducted both *in vivo* and *ex vivo* to confirm and to explore the underlying mechanisms, such as why cardiomyocyte hypoxia/reoxygenation leads to increased AMPK activation with concomitant reductions in STAT3 activation but a further increase in AMPK activation induced by HPo and/or by APN-increased STAT3 activation [41].

## 5. Conclusions

**Figure 6:**
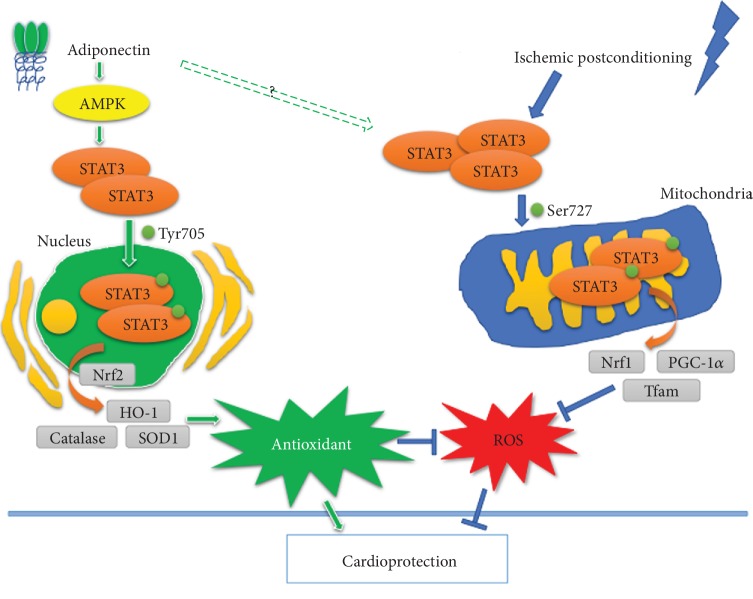
Schematic diagram of proposed signaling involved in adiponectin and ischemic postconditioning protection against myocardial ischemia-reperfusion injury via AMPK-dependent and AMPK-independent STAT3 activation. Adiponectin (APN) on the one hand activates AMPK thereby activates STAT3, which increases STAT3 nuclear intension. This enhanced nuclear STAT3 promotes antioxidant gene expression and enhances antioxidant capacity. On the other hand, APN activates STAT3 and increases its mitochondrial intention, which subsequently increases Nrf1 and PGC-1*α*, thereby reduces reactive oxygen species (ROS) production. These APN-induced increase of antioxidant capacity and reduction of ROS production result in attenuation of myocardial ischemia-reperfusion (IR) injury. While ischemic postconditioning (IPo) activates STAT3 and increases its mitochondrial intension thereby enhances Nrf1 and PGC-1*α*, it reduces ROS production and eventually attenuates myocardial IR injury. Combinational use of APN and IPo, through activating STAT3, increases its nuclear and mitochondrial retentions, which concomitantly enhances antioxidant capacity and reduces ROS production, jointly lead to attenuation of myocardial IR injury.

In summary, as illustrated in Figure 6, our study demonstrated that APN should have conferred cardioprotection by enhancing antioxidant capacity through activating STAT3 at Tyr705 which is AMPK-dependent and by reducing ROS production through activating STAT3 at Ser727 which is AMPK-independent. By contrast, IPo conferred cardioprotection by reducing ROS production through AMPK-independent activation of STAT3 at Ser727. Combinational use of APN and IPo conferred synergistic cardioprotective effects against myocardial IR injury. However, caution should be exercised when choosing the dose and regimen of exogenous APN to avoid the occurrence of hyperadiponectinemia and the subsequent potential adverse effects, as IPo per se could also enhance endogenous adiponectin. Also, it should be noted that adenoviral-mediated APN expression may not be an ideal model of physiological relevance, but the insight gained from this mechanistic exploratory study may serve to stimulate the use of drugs that can enhance endogenous APN secretion as an adjuvant therapy to increase the effectiveness of ischemic postconditioning in clinical settings.

## Figures and Tables

**Table 1:** Hemodynamic measurements at baseline and 2 hours of reperfusion in rats with sham and myocardial ischemia-reperfusion with or without adiponectin and ischemic postconditioning.

	Sham	IR	IR+APN	IR+IPo	IR+APN+IPo
Baseline					
Heart rate (min^−1^)	280 ± 10	279 ± 7	276 ± 10	281 ± 10	277 ± 9
MAP (mmHg)	115 ± 3	113 ± 5	116 ± 5	112 ± 5	110 ± 6
RPP (mmHg·min^−1^/10000)	33 ± 4	36 ± 3	34 ± 4	37 ± 3	38 ± 2

2 hours of reperfusion					
Heart rate (min^−1^)	287 ± 9^#^	155 ± 8^∗^	203 ± 6^∗^^#^	197 ± 7^∗^^#^	197 ± 9^∗^^#^
MAP (mmHg)	113 ± 4^#^	62 ± 8^∗^	88 ± 4^∗^^#^	88 ± 4^∗^^#^	91 ± 3^∗^^#^
RPP (mmHg·min^−1^/10000)	36 ± 2^#^	18 ± 1^∗^	26 ± 1^∗^^#^	26 ± 2^∗^^#^	27 ± 1^∗^^#^

Rats were subjected to 30 min of coronary occlusion and 2 hours of reperfusion (IR) with or without adiponectin (APN, tail vein-injected 7 days before inducing IR) or ischemic postconditioning (IPo) or their combination (APN+IPo). All values are mean ± SEM. *n* = 8 animal *per* group. Heart rate, mean arterial pressure (MAP), and rate pressure product (RPP) were measured at baseline and 2 hours of reperfusion. ^∗^*p* < 0.05 or *p* < 0.01*vs.* their corresponding baseline, ^#^*p* < 0.05 or *p* < 0.01*vs.* their corresponding IR.

## Data Availability

The data used to support the findings of this study are included in the article.
